# Immune profiling and prognostic model of pancreatic cancer using quantitative pathology and single-cell RNA sequencing

**DOI:** 10.1186/s12967-023-04051-4

**Published:** 2023-03-21

**Authors:** Kai Chen, Qi Wang, Xinxin Liu, Xiaodong Tian, Aimei Dong, Yinmo Yang

**Affiliations:** 1 Department of General Surgery, Peking University First Hospital, 8th Xishiku Street, Beijing, 100034 China; 2 Department of Endocrinology, Peking University First Hospital, 8th Xishiku Street, Beijing, 100034 China

**Keywords:** Immune profiles, Pancreatic cancer, scRNA-seq, Prognosis, Quantitative pathology

## Abstract

**Background:**

Pancreatic ductal adenocarcinoma (PDAC) has a complex tumor immune microenvironment (TIME), the clinical value of which remains elusive. This study aimed to delineate the immune landscape of PDAC and determine the clinical value of immune features in TIME.

**Methods:**

Univariable and multivariable Cox regression analyses were performed to evaluate the clinical value of immune features and establish a new prognostic model. We also conducted single-cell RNA sequencing (scRNA-seq) to further characterize the immune profiles of PDAC and explore cell-to-cell interactions.

**Results:**

There was a significant difference in the immune profiles between PDAC and adjacent noncancerous tissues. Several novel immune features were captured by quantitative pathological analysis on multiplex immunohistochemistry (mIHC), some of which were significantly correlated with the prognosis of patients with PDAC. A risk score-based prognostic model was established based on these immune features. We also constructed a user-friendly nomogram plot to predict the overall survival (OS) of patients by combining the risk score and clinicopathological features. Both mIHC and scRNA-seq analysis revealed PD-L1 expression was low in PDAC. We found that PD1 + cells were distributed in different T cell subpopulations, and were not enriched in a specific subpopulation. In addition, there were other conserved receptor-ligand pairs (CCL5-SDC1/4) besides the PD1-PD-L1 interaction between PD1 + T cells and PD-L1 + tumor cells.

**Conclusion:**

Our findings reveal the immune landscape of PDAC and highlight the significant value of the combined application of mIHC and scRNA-seq for uncovering TIME, which might provide new clues for developing immunotherapy combination strategies.

**Supplementary Information:**

The online version contains supplementary material available at 10.1186/s12967-023-04051-4.

## Introduction

Pancreatic cancer is one of the most aggressive gastrointestinal cancers with an overall 5 year survival rate of 11% [1]. Pancreatic ductal adenocarcinoma (PDAC) is the most common histological type of pancreatic cancer. Radical resection is the only potential curative treatment for PDAC. However, approximately 50% of patients have advanced PDAC at the time of diagnosis and have lost the chance to undergo surgery. Even after radical resection, most patients relapse within 2 years [2]. Multidisciplinary management, positive genomic testing, and integrated supportive care are recommended for all patients with PDAC [3]. Despite the continuous introduction of new regimens, drugs available to significantly improve the prognosis of patients with PDAC remain absent. Recently, a growing body of evidence has demonstrated that immune checkpoint inhibitors (ICIs) showed good treatment outcomes in non-small cell lung cancer, melanoma, and colorectal cancer [4–8], but not in PDAC. The common targets of ICIs include cytotoxic T lymphocyte protein 4 (CTLA-4), programmed death-1 (PD1), and programmed death-ligand 1 (PD-L1). The mechanism by which ICIs exert their therapeutic effect is that the blockade of immune checkpoints releases brake signals to promote the endogenous anti-tumor immune response. However, the reason why ICIs fail in PDAC is largely unknown. A comprehensive delineation of immune profiles of PDAC is fundamental for improving ICIs insensitivity. Moreover, the clinical value of immune checkpoint molecules remains to investigated.

Pancreatic cancer is characterized with a complex tumor immune microenvironment (TIME). In comparison with conventional immunohistochemistry (IHC), multiplex IHC (mIHC) can detect the expression of multiple markers simultaneously in situ, thereby identifying the phenotype of each cell and cell-to-cell spatial interaction in the tissue, and in combination with quantitative pathology, highly reproducible statistical data can be obtained [9]. This method is well-suited for delineating the complicated TIME in PDAC. Recently, single-cell RNA sequencing (scRNA-seq) has demonstrated a wide range of applications that has been used to uncover the cell landscapes of various tumor tissues. The transcriptional profile of each cell was obtained by scRNA-seq analysis, consisting of single cell capture, cDNA library preparation, RNA sequencing, and data mining [10]. Immune cell composition and receptor-ligand interactions between different cell types can be easily identified using scRNA-seq. Currently, the immune profiles and cell-to-cell interactions between PD1 + tumor infiltrating T cells and PD-L1 + tumor cells in PDAC have not been studied at the single-cell levels.

Here, we applied mIHC and quantitative pathology to characterize the immune profiles of PDAC and capture novel immune features related to immune checkpoint molecules. The univariable Cox regression (UniCox) and multivariable Cox regression (MultiCox) analyses were performed to evaluate the clinical value of these immune features and develop a new risk score-based prognostic model. Furthermore, a user-friendly nomogram plot was drawn by combining the risk score and clinicopathological features, which had a good performance in predicting the overall survival of patients with PDAC. ScRNA-seq was also conducted to verify the results of mIHC and identify new receptor-ligand pairs between PD1 + T cells and PD-L1 + tumor cells in PDAC. Thus, our findings highlight the significance of the combined application of mIHC and scRNA-seq for uncovering the immune landscape of PDAC.

## Materials and methods

### Human samples and study design

All H&E stained slides from eighty patients from September 2020 to January 2022 were reviewed for confirming PDAC diagnosis by a pathologist. The corresponding tumor and adjacent noncancerous areas were carefully marked. Duplicated 1.5 mm diameter tissue cores were selectively punched and transferred to recipient tissue array blocks. The tissue microarray (TMA) was set up according to the instruction reported in previous study [11]. For scRNA-seq analysis, a total of six PDAC and six adjacent noncancerous resection specimens were obtained from the Department of General Surgery at Peking University First Hospital. All patients with PDAC did not receive any treatments before collecting specimens. This study was reviewed and approved by the Ethics Committee of Peking University First Hospital (Approval No. 2020–352). Written informed consent was signed prior to acquisition of tissue from all patients. All experiments were conducted in accordance with ethical guidelines of the Declaration of Helsinki.

**Fig. 1:**
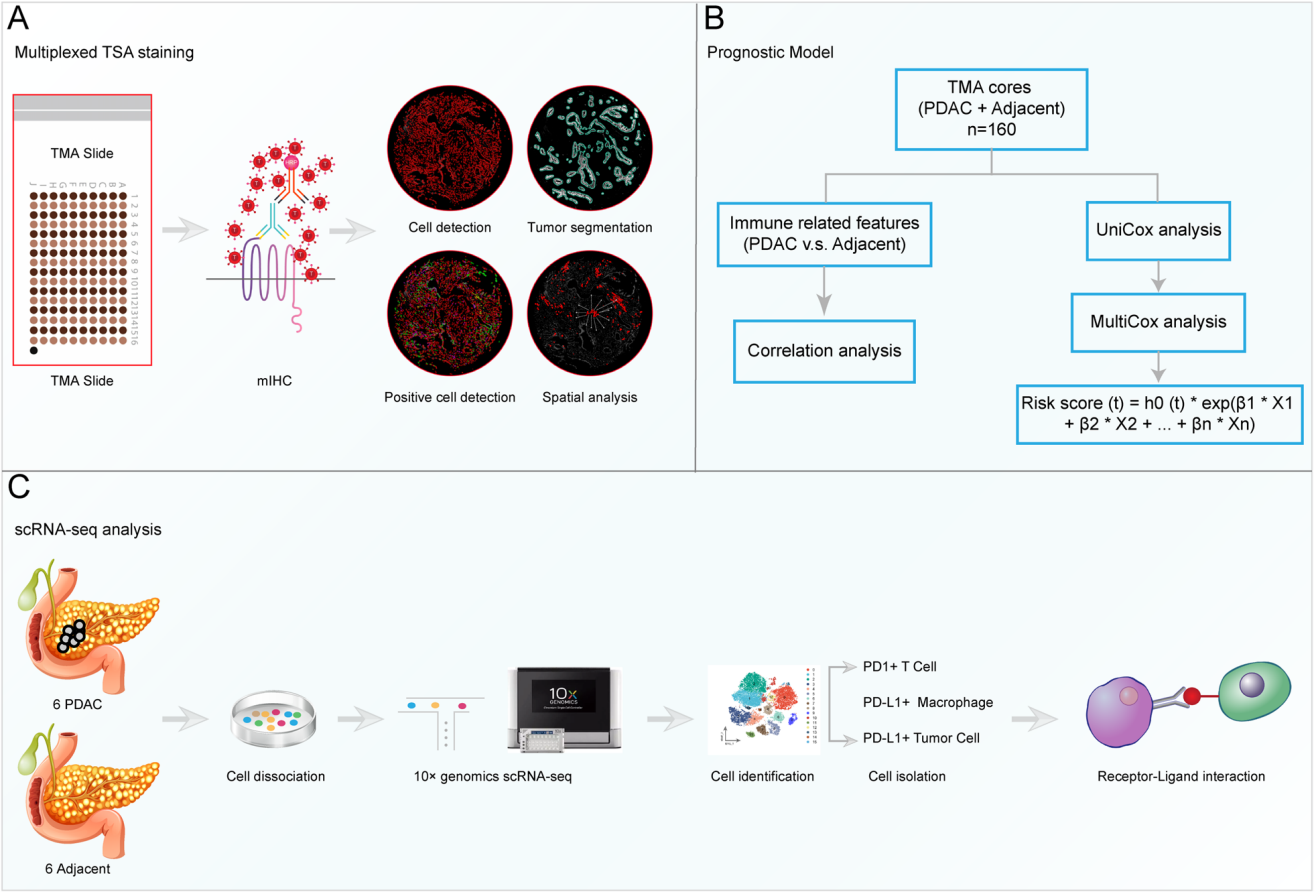
Graphical scheme describing the workflow. **A** TMA was subjected to multiplex IHC (mIHC), and the immune features were generated using quantitative pathology to characterize the immune landscape of PDAC. **B** We compared the differences in immune features between PDAC and adjacent noncancerous TMA cores. The correlations between these immune features were observed. Then, the prognostic models were developed using Univariable and Multivariable Cox regression according to these immune features. **C** ScRNA-seq was conducted to further explore the immune landscape of PDAC and identify the receptor-ligand interactions between PD1 + T cells and PD-L1 + tumor cells

As showed in Fig. 1, this study included three parts. First, mIHC was conducted and immune features were captured using quantitative pathology to characterize the immune landscape of PDAC. Second, Univariable and Multivariable Cox regression were performed to evaluate the clinical values of these immune features and develop a new prognostic model. Finally, we conducted scRNA-seq analysis to further verify the results of mIHC. The malignant ductal cells were identified using the inferCNV analysis. In addition, we explored cell-to-cell communication between PD1 + T cells and PD-L1 + tumor cells.

### Multiplex immunohistochemistry

To explore the expression and distribution of Pan-CK, CD68, PD1, PD-L1, and CD8 in PDAC, TMA sections from PDAC and adjacent noncancerous tissues were subjected to multiplex immunohistochemistry (mIHC) using PANO Multiplex IHC kit (Panovue, Cat. No. 10234100100) according to manufacturer’s instructions. Briefly, TMA section was incubated in hot-air oven at 65 °C overnight, deparaffinized in fresh xylene for 10 min three times, rehydrated in graded ethanol (100–95–70%), then washed three times with PBS. The antigen retrieval was carried out with microwave heating method and cooled down for at least 15 min in the ice water bath. After blocking in blocking solution (Panovue, Cat. No. 0018001120) for 15 min at room temperature, TMA section was incubated with primary antibody for 30 min, secondary antibody for 10 min, and TSA Opal fluorophores for 10 min. Repeated antigen retrieval, blocking, primary and antibody incubation, TSA Opal fluorophores staining for each marker. Finally, TMA section was counterstained with DAPI (Sigma-Aldrich, Cat. No. D9542) for 5 min and mounted. Whole TMA section imaging was conducted using the panoVIEW VS200 (china). The following primary antibodies were used. Pan-CK (Abcam, Cat. no. ab7753, 1:200), CD68 (Abcam, Cat. no. ab192847, 1:300), PD1 (Abcam, Cat. no. ab237728, 1:500), PD-L1 (Abcam, Cat. no. ab237726, 1:400), and CD8 (CST, Cat. no. 85336, 1:100).

### Quantitative pathology

The QuPath software (v.0.3.2) was downloaded (https://qupath.github.io/). The proportion and density of positive cells and spatial analysis were performed according to a standard protocol, as described on the website above. In brief, a new project was created and scanning image of TMA was imported into QuPath. The image type of fluorescence was selected. Adjusted each fluorescence channel using the Brightness/Contrast command. TMA dearrayer was performed with TMA core diameter of 1.7 mm and adjusted TMA grid manually. Tissue segmentation was conducted to classify each TMA core into tumor and stromal area according to the expression of PanCK using the pixel classification tool. Cell detection was completed by setting the optimal threshold of DAPI. Next, CD8 + and PD1 + cells were detected by single measurement classifier. For CD68 + cells and PD-L1 + regions that were hard to be detected only by the threshold of fluorescence, we employed machine learning algorithm to complete automatic detection by training the object or pixel classifier based on annotations. Created composite classifier to detect double/triple positive cells. Finally, the spatial analysis was conducted. We determined the density of immune cells 30/50 μm away from tumor region and PD1 + cells 30/50 μm away from PD-L1 + region. Of note, all features in the tumor and stromal regions were calculated respectively. The abbreviations of all immune features used in this study were listed below: proportion of stromal cells (P_sc, %), proportion of stromal area (P_sa, %), density of CD8 + cells (D_cd8, cells/mm^2), density of PD1 + cells (D_pd1, cells/mm^2), density of CD68 + cells (D_cd68, cells/mm^2), proportion of total PD-L1 + cells (P_topdl1, %), proportion of tumor PD-L1 + cells (P_tupdl1, %), proportion of stromal PD-L1 + cells (P_spdl1, %), density of CD8 + PD1 + cells (D_cd8_pd1, cells/mm^2), distance_PDL1_PD1_30 μm /Total area (D_pdl1_30_pd1, n/mm^2), distance_Tumor_immune_30 μm /Total area (D_tu_30_im, n/mm^2), distance_Tumor_CD8A_30 μm /Total area (D_tu_30_cd8, n/mm^2), distance_Tumor_CD68_30 μm /Total area (D_tu_30_cd68, n/mm^2), distance_PDL1_PD1_50 μm /Total area (D_pdl1_50_pd1, n/mm^2), distance_Tumor_immune_50 μm /Total area (D_tu_50_im, n/mm^2), distance_Tumor_CD8A_50 μm /Total area (D_tu_50_cd8, n/mm^2), distance_Tumor_CD68_50 μm /Total area (D_tu_50_cd68, n/mm^2).

### Construction of prognostic model

Immune features generated by mIHC and clinicopathologic data were imported into R software (v4.0.3). Univariable and Multivariable Cox regression were performed to evaluate the clinical values of these features using ‘survival’ R package (v3.2.7). Risk score = h0 × e^∑_i = 0_^n^ exp (). Patients were classified into two groups (high vs. low risk) according to the median risk score. The KM and ROC curves were used to evaluate the value of prognostic models using ‘survivalROC’ R package (v1.0.3). Then, a nomogram was developed to predict the overall survival of patients with PDAC by combining the immune and clinicopathologic features using ‘rms’ R package (v6.2.0). The calibration curve was also drawn to evaluate the accuracy of nomogram-predicted patients’ survival.

### Tissue dissociation and cell purification

All fresh resection specimens were preserved in the tissue storage solution (Miltenyi, Cat. No. 130-100-008) on ice and transported to the laboratory in CapitalBio Technology company within 1.5–2 h. To ensure the success of tissue dissociation, multiple resection specimens from the same patients were digested. All specimens were cut into around 1 mm pieces and incubated in an optimal digestive solution, including enzyme cocktail, consisting of the Type VIII Collagenase (Sigma-Aldrich, Cat. No. C2139), DNase I (Sigma-Aldrich, Cat. no. D5025), trypsin inhibitor (Sigma-Aldrich, Cat. No. T6522), and Dispase II (neutral protease, grade II, Sigma-Aldrich, Cat. No. 4942078001). The single cell suspension was filtered with a 40 μm cell strainer (BD, Cat. No. 352340), then incubated with red blood cell lysis buffer (Roche, Cat. no. 11814389001) at 4 °C for 10 min.

### cDNA library preparation

The concentration of single cell suspension was determined using the Count Star instrument and adjusted to 1000 cells/μl. The cell suspension was loaded onto the Chromium single cell controller (10 × Genomics) to generate single-cell gel beads in the emulsion using single cell 3’ Library and Gel Bead Kit v3.1 (10 × Genomics, Cat. No. 1000121) and Chromium Next GEM Chip G Single Cell Kit (10 × Genomics, Cat. No. 1000120) according to the manufacturer’s instructions. Briefly, cells were suspended in PBS containing 0.04% BSA. About 6,000 cells were loaded to each channel, and the target cell will be captured about 3000 cells per channel. Captured cells were lysed and the released RNA were barcoded by reverse transcription in each GEM. Reverse transcription was completed in 200 μl tubes (NEST Biotechnology, Cat. No. 401001) on a S1000TM Touch Thermal Cycler (Bio Rad) at 53 °C for 45 min, then at 85 °C for 5 min, and hold at 4 °C. The cDNA libraries were generated, amplified, and quality assessed using the Agilent 4200. Finally, the cDNA libraries were sequenced using an Illumina Novaseq6000 sequencer with a sequencing depth of at least 100,000 reads per cell with paired-end 150 bp (PE150) reading strategy.

### ScRNA-seq data mining and quality control

Raw data (BCL files) from Illumina Novaseq6000 platform was converted to fastq files using Illumina-implemented software bcl2fastq (v2.19.0.316). cDNA reads were aligned to human reference genome (GRCh38). Low-quality cells and genes filtering, barcode and UMI counting were performed with the cellranger software (v6.1.2) to obtain the filtered gene-cell matrixes. Next, gene-cell matrixes were imported into R software to further filtered out low-quality cells (< 500 genes/cell, > 25% mitochondria genes, < 1000 transcripts/cell) and genes (< 10 cells/gene) using ‘Seurat’ R package (v3.2.3). Gene expression levels were normalized (LogNormalize) with “NormalizedData” function. A total of 2000 highly variable genes were selected and used to conduct PCA reduction dimension. The t-distributed stochastic neighbor embedding (t-SNE) was performed. The ‘Soupx’ R package was applied to reduce the ambient mRNA contamination. Doublets were identified using the ‘DoubletFinder’ R package (v2.0.3), assuming that it was around 5% doublet formation rate to the loaded cells per specimen in a droplet channel. In addition, the ‘Harmony’ R package (https://github.com/immunogenomics/harmony) was used to integrate gene-cell matrixes derived from different specimens. We identified each cell cluster by matching the cluster-specific genes with known signatures of cell populations reported in previous studies and CellMarker database.

### Single-cell CNV inferring

Somatic large-scale chromosomal copy number variation (CNV) was inferred using ‘inferCNV’ R package (v1.6.0). In brief, a new gene-cell matrix of ductal cells, annotation data, and gene/chromosome position files were prepared. Both T cells and macrophages were taken as reference cells as they are considered to have no CNV. The CNV score of each cluster was calculated as quadratic sum of CNV region.

### GO analysis

The differentially expressed genes (DEGs) were identified using the FindMarkers function in ‘Seurat’ R package. The online tool g: Profiler (https://biit.cs.ut.ee/gprofiler/gost) was applied to conduct GO analysis for top 80 DEGs between the two groups.

### Receptor-ligand interaction

A new gene-cell matrix of PD1 + T cells and PD-L1 + macrophages and tumor cells was constructed and imported into R software. The receptor-ligand pairs were identified using R package ‘iTALK’ (v0.1.0) (https://github.com/Coolgenome/iTALK) with default parameters. Considering the test efficiency and computational burden, 200–500 cells of each cell cluster were randomly selected for cell-to-cell interaction analysis. The visualization of receptor-ligand pairs was divided into four groups, including growth factors, cytokines, immune checkpoints, and others.

### Statistical analysis

All statistical analyses were conducted using the SPSS (v22.0) and R software. Univariable and Multivariable Cox regression were performed to develop the prognostic models. The KM method and the corresponding log-rank test were performed to evaluate the prognostic value of risk score-based prognostic models. The area under the ROC curve was calculated to assess the sensitivity and specificity of the model. For continuous variable, the independent-samples t test and Mann–Whitney U test was performed to compare means between two groups. For categorical variable, the chi-square test or rank sum test was performed. Coefficients of Spearman’s rank correlation or Pearson’s correlation were calculated to describe the correlation of two variables. Statistical significance was defined as ∗ P < 0.05, ∗  ∗ P < 0.01, and ∗  ∗  ∗ P < 0.001.

## Results

### Clinical characteristics of PDAC patients in TMA

To explore the TIME and construct a prognostic model for PDAC, we conducted mIHC using TMA and quantitative pathology analysis. The general characteristics of patients with TMA were shown in Additional file 2: Table S1. A total of 160 TMA cores, including 80 tumor and paired 80 adjacent noncancerous pancreatic cores, were stained. After filtering out low-quality cores, 66 pairs of cores were included in the subsequent analysis. As summarized in Table 1, all patients (n = 66) had a confirmed diagnosis of PDAC. Twenty-eight (42.42%) patients were diagnosed with stage I PDAC, 16 (24.24%) with stage II, 10 (15.15%) with stage III, and 12 (18.18%) with stage IV. There were 44 (66.67%) patients had tumors in the head of the pancreas and 22 (33.33%) in the body/tail. None of the patients received any treatment prior to sample acquisition; 10 (15.15%) had vascular invasion, and 11 (16.67%) had distant metastases.

**Table 1:** Clinical characteristics of patients in prognostic model

	Total	Model 1	High risk	P_value	Model 2	High risk	P_value
		Low risk			Low risk		
PDAC patients (n)	66	33	33	–	33	33	–
Sex (F [%])	35 (53.03)	21 (63.64)	14 (21.21)	0.084	21 (63.64)	14 (21.21)	0.084
Age (years)	63.27 ± 10.21	63.73 ± 9.35	62.82 ± 11.14	0.721	65.30 ± 9.88	61.24 ± 10.28	0.107
Grade (n [%])							
1	9 (13.64)	6 (18.18)	3 (9.09)	0.609	6 (18.18)	3 (9.09)	0.233
2	21 (31.82)	9 (27.27)	12 (36.36)		11 (33.33)	10 (30.30)	
3	36 (54.55)	18 (54.55)	18 (54.55)		16 (48.48)	20 (60.61)	
TNM stage (n [%])							
I	28 (42.42)	17 (51.52)	11 (33.33)	0.177	17 (51.52)	11 (33.33)	0.359
II	16 (24.24)	9 (27.27)	7 (21.21)		8 (24.24)	8 (24.24)	
III	10 (15.15)	4 (12.12)	6 (18.18)		3 (9.09)	7 (21.21)	
IV	12 (18.18)	3 (9.09)	9 (27.27)		5 (15.15)	7 (21.21)	
Tumor site (n [%])							
Head	44 (66.67)	20 (60.61)	24 (72.73)	0.296	18 (54.55)	26 (78.79)	0.068
Body/Tail	22 (33.33)	13 (39.39)	9 (27.27)		15 (45.45)	7 (21.21)	
CA 19–9 (U/ml)	197.15 (68.59–884.13)	173.20 (44.80–821.15)	199.30 (69.08–1000.00)	0.657	212.20 (48.71–821.15)	195.00 (68.58–944.80)	0.944
Vascular invasion (n [%])	10 (15.15)	6 (18.18)	4 (12.12)	0.492	5 (15.15)	5 (15.15)	1
Intravascular cancer embolus (n [%])	21 (31.82)	9 (27.27)	12 (36.36)	0.428	8 (24.24)	13 (39.39)	0.186
Perineural invasion (n [%])	19 (28.79)	9 (27.27)	10 (30.30)	0.786	9 (27.27)	10 (30.30)	0.786
Peripancreatic infiltarion (n [%])	48 (72.73)	21 (63.64)	27 (81.82)	0.097	22 (66.67)	26 (78.79)	0.269
Distant metastasis (n [%])	11 (16.67)	3 (9.09)	8 (24.24)	0.099	4 (12.12)	7 (21.21)	0.322
Lymph node metastasis (n [%])	26 (39.39)	12 (36.36)	14 (42.42)	0.614	11 (33.33)	15 (45.45)	0.314
Maximum diameter (cm)	3.5 (3.0–4.5)	3.5 (3.0–4.25)	3.5 (2.25–4.5)	0.746	3.5 (3.0–4.5)	3.5 (2.25–4.25)	0.902

### Differences in immune profiles between PDAC and adjacent noncancerous pancreas

**Fig. 2:**
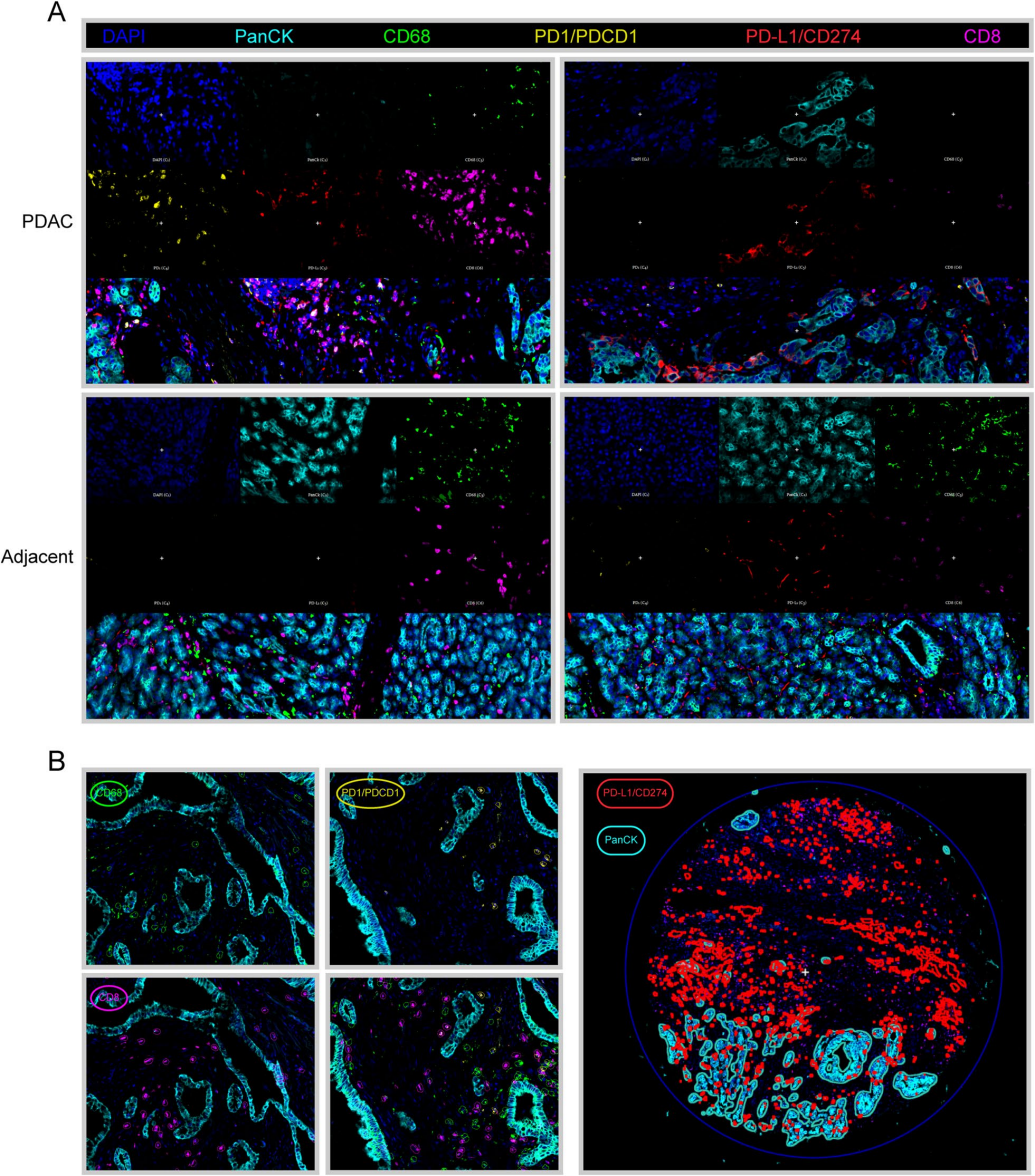
Immune landscape of PDAC. **A** Representative images of mIHC in PDAC and adjacent noncancerous TMA cores, which were exported from Qupath software. Cell markers, including PanCK, CD68, PD1, PD-L1, and CD8 were labeled with different colors. **B** Representative images of quantitative pathological analysis. Tumor and stromal regions were separated according to PanCK expression. The PD1 + /CD8 + cells were identified by single measurement classifier, while CD68 + /PD-L1 + cells were identified by training the object or pixel classifier

**Fig. 3:**
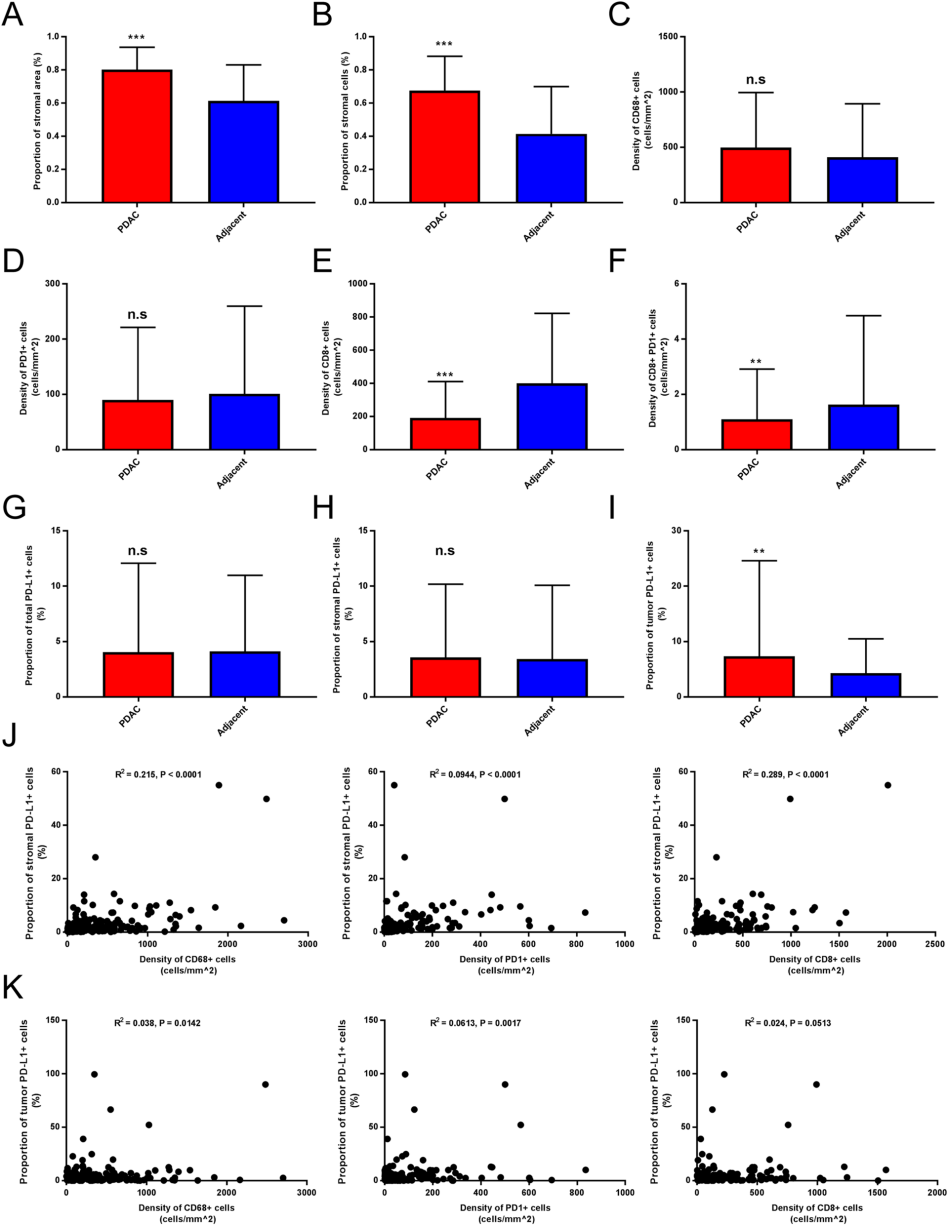
Differences in immune profile between PDAC and adjacent noncancerous tissues. **A**–**B** Proportion of stromal cells and area were compared between two groups. **C**–**F** Density of immune markers were compared. **G**–**I** The PD-L1 expression was compared between two groups. **J**–**K** Correlation analysis was performed between stromal/tumor PD-L1 expression and density of CD68 + /PD1 + /CD8 + cells

The mIHC was used to examine specific cell markers, including PanCK, CD68, PD1, PD-L1, and CD8 (Fig. 2A). The entire core was divided into tumor and stromal areas according to the expression of PanCK. The immune-related features of each core were captured using the Qupath software (Fig. 2B, Additional file 3: Table S2). There were significantly higher proportions of stromal area and cells in the PDAC cores than in the adjacent noncancerous pancreatic cores (Fig. 3A–B). No significant differences were detected in the densities of CD68 + and PD1 + cells between the two groups (Fig. 3C–D). Compared to the adjacent noncancerous pancreas, PDAC had a lower density of CD8 + and CD8 + PD1 + cells (Fig. 3E–F). In addition, we compared PD-L1 expression between the two groups. The results showed that there were no significant differences in the proportions of total and stromal PD-L1 + cells, while a higher proportion of tumor PD-L1 + cells was detected in the PDAC group (Fig. 3G–I).

A growing body of evidence supported the correlation between PD-L1 expression and other immune features [12–14]. In pancreatic tissue, there was a significant correlation between the proportion of stromal/tumor PD-L1 + cells and the density of CD68 + /PD1 + cells. Interestingly, the proportion of PD-L1 + cells in the stromal area, rather than in the tumor area, correlated with the density of CD8 + cells (Fig. 3J–K).

### Development of prognostic model based on immune-related features

**Fig. 4:**
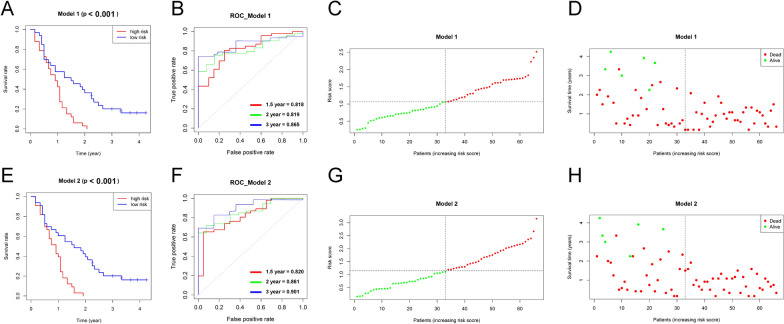
Prognostic models of PDAC were developed according to the immune features. **A**–**D** Multivariable Cox regression was conducted to develop prognostic model 1. The KM plot showing different OSs between patients in the high and low risk score groups (**A**). The ROC curve was employed to evaluate the accuracy of prognostic model for 1.5 year, 2 year, and 3 year OSs (**B**). The distribution of risk score for each patient. High and low risk groups were separated by dashed, labeled with red and green colors, respectively (**C**). The scatter plot showing survival status of each patient (**D**). **E**–**H** The prognostic model 2 was showed

Intra-tumor cytotoxic T cell, macrophage populations, and PD-L1 expression have been proven to be powerful candidate biomarkers for predicting the prognosis and immune checkpoint treatment response [15–17]. In addition to counting the proportion and density of positive cells, we also performed a spatial analysis for PDAC cores. The distances from the selected cells to the tumor and PD-L1 annotations were calculated (Additional file 2: Table S2). UniCox analysis was performed and we found that P_sc, D_cd8, D_pd1, D_cd8_pd1, D_pdl1_30_pd1, D_tu_30_cd8, D_pdl1_50_pd1, and D_tu_50_cd8 were significantly associated with the OS (Table 2). Next, MultiCox analysis was employed to construct risk prognostic models based on immune-related features. The risk score for each subject was calculated as follow: Model 1: Risk score (t) = h0 (t) × exp (P_sc × 2.121 + D_tu_50_cd8 × 0.237); Model 2: Risk score (t) = h0 (t) × exp (P_sc × 6.831—P_sa × 8.088—D_pd1 × 0.679—P_topdl1 × 0.260 + D_pdl1_50_pd1 × 0.759 + D_tu_50_cd8 × 0.245). All subjects were divided into low- and high-risk groups according to the median cutoff value of the risk scores. The demographic characteristics of the patients in the two groups were comparable (Table 1). Kaplan–Meier (KM) curves showed that the subjects in the low-risk groups had significantly longer OS than those in the high-risk groups (Model 1: p < 0.001; Model 2: p < 0.001) (Fig. 4A, E). A time-dependent receiver operating characteristic curve (ROC) was used to evaluate the accuracy of predicting the 1.5 year, 2 year, and 3 year OSs. The area under curve (AUC) values were 0.818, 0.819, and 0.865 in Model 1 and 0.820, 0.861, and 0.901 in Model 2 (Fig. 4B, F). As the risk score increased, the prognoses of the subjects worsen (Fig. 4C, D, G–H).

**Table 2:** Univariable and multivariable cox regression analysis of prognostic features

Features	UniCox		P_value	MultiCox_model 1		P_value	MultiCox_model 2		P_value
	HR	95% CI		HR	95% CI		HR	95% CI	
P_sc	5.466	1.302–22.938	**0.02**	8.340	1.771–39.281	**0.007**	9.257	10.435–82121.170	**0.003**
P_sa	7.823	0.682–89.723	0.098				3.073	1.804E-07–0.523	**0.033**
D_cd8	1.239	1.048–1.464	**0.012**						
D_pd1	1.152	1.000–1.327	**0.049**				5.069	0.300–0.856	**0.011**
D_cd68	1.071	0.901–1.274	0.435						
P_topdl1	1.015	0.828–1.245	0.883				7.710	0.565–1.053	0.102
P_tupdl1	0.992	0.850–1.158	0.92						
P_spdl1	1.077	0.865–1.340	0.508						
D_cd8_pd1	1.158	1.021–1.313	**0.023**						
D_pdl1_30_pd1	1.152	1.004–1.322	**0.044**						
D_tu_30_im	1.065	0.904–1.254	0.453						
D_tu_30_cd8	1.182	1.003–1.393	**0.046**						
D_tu_30_cd68	1.021	0.899–1.161	0.746						
D_pdl1_50_pd1	1.163	1.010–1.339	**0.035**				2.136	1.216–3.753	**0.008**
D_tu_50_im	1.099	0.929–1.301	0.271						
D_tu_50_cd8	1.217	1.034–1.432	**0.018**	1.268	1.072–1.49	**0.006**	1.278	1.028–1.587	**0.027**
D_tu_50_cd68	1.037	0.911–1.180	0.58						

The bold *P* value means *P* < 0.05 with significantly statistical difference

UniCox, Univariable Cox regression, MultiCox, Multivariable Cox regression; *HR*, hazard ratio, *CI* confidence interval. Model 1: variables with P_value < 0.05 were used; Model 2: all variables were used

### Nomogram for predicting the prognosis of patients with PDAC

**Fig. 5:**
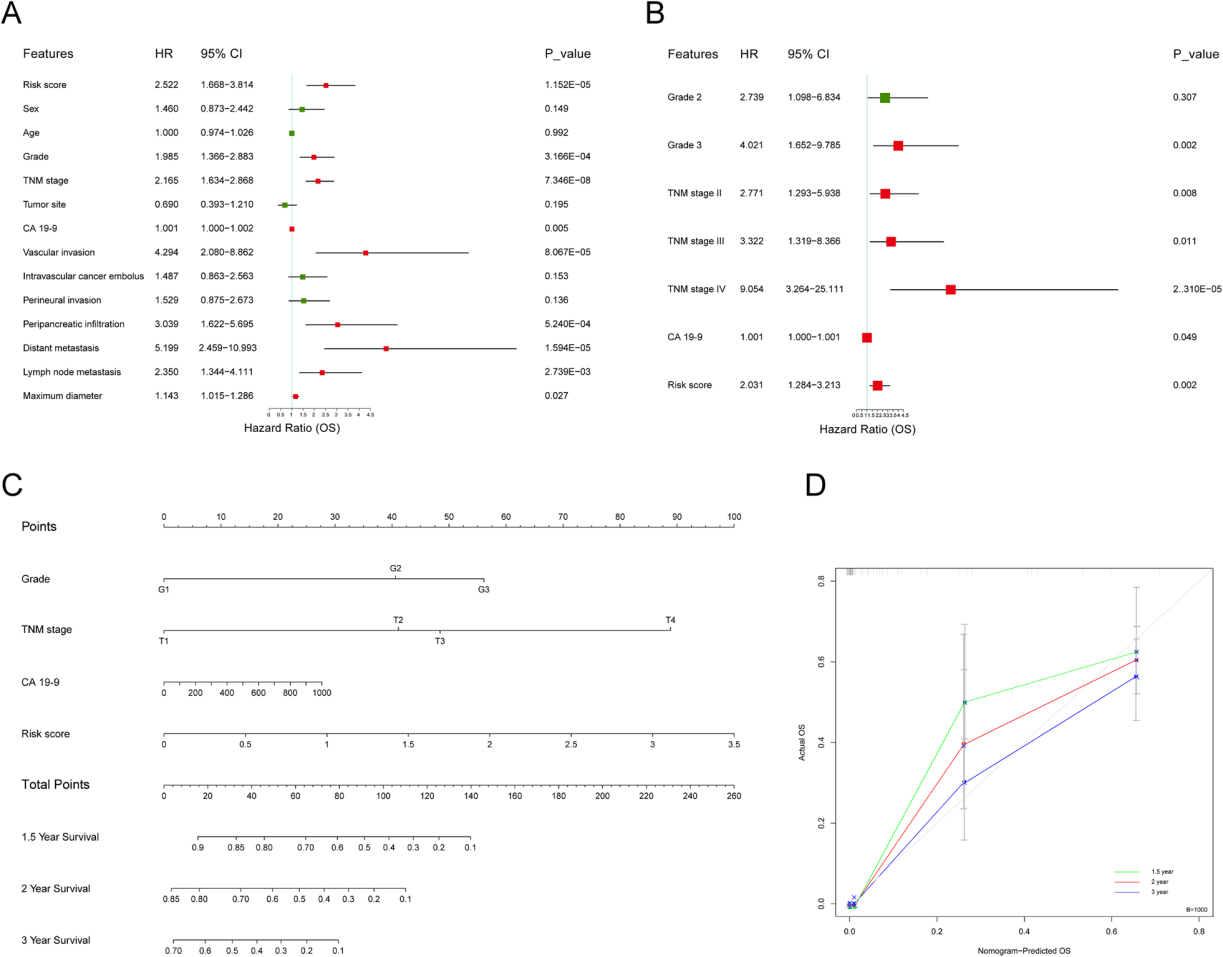
Nomogram to predict the survival outcome of patients with PDAC. **A**–**B** Univariable and Multivariable Cox regression were conducted to find prognostic factors of OS in PDAC, red boxes represent P < 0.05 in the forest plots. The clinicopathological features and risk score were analyzed. **C** The nomogram was drawn to predict 1.5 year, 2 year, and 3 year Oss for PDAC. The higher total point was, the lower survival rates patients will have. **D** Calibration curve was used to show agreement between actual and nomogram-predicted OSs, the gray diagonal line represented reference line

The clinicopathological features of the patients were collected, and UniCox and MultiCox analyses were performed. The UniCox result showed that risk score [hazard ratio (HR): 2.522, 95% confidence interval (CI): 1.668–3.814, p < 0.001], grade [HR: 1.985, 95% CI 1.366–2.883, p < 0.001], TNM stage [HR: 2.165, 95% CI 1.634–2.868, p < 0.001], CA 19–9 [HR: 1.001, 95% CI 1.000–1.002, p = 0.005], vascular invasion [HR: 4.294, 95% CI 2.080–8.862, p < 0.001], peripancreatic infiltration [HR: 3.039, 95% CI 1.622–5.695, p < 0.001], distant metastasis [HR: 5.199, 95% CI 2.459–10.993, p < 0.001], lymph node metastasis [HR: 2.350, 95% CI 1.344–4.111, p = 0.003], and maximum diameter [HR: 1.143, 95% CI 1.015–1.286, p = 0.027] were significantly correlated to the OS of patients (Fig. 5A). Furthermore, MultiCox analysis indicated that the risk score [HR: 2.031, 95% CI 1.284–3.213, p = 0.002], in addition to grade 3, TNM stage, and CA 19–9, was an independent prognostic factor for PDAC (Fig. 5B). Therefore, the risk score calculated according to immune features using mIHC is a promising prognostic biomarker for PDAC.

An easy-to-use and clinically adaptable nomogram was constructed to predict the OS of patients by combining the variables of risk score, grade, TNM stage, and CA 19–9. As depicted in Fig. 5C, patients with higher total points were predicted to have lower 1.5 year, 2 year, and 3 year OS rates. The calibration curve showed good consistency between the actual OSs and nomogram predicted OSs, suggesting the accuracy of this nomogram (Fig. 5D).

### ScRNA-seq delineates cellular landscape of PDAC and adjacent noncancerous pancreatic tissues

**Fig. 6:**
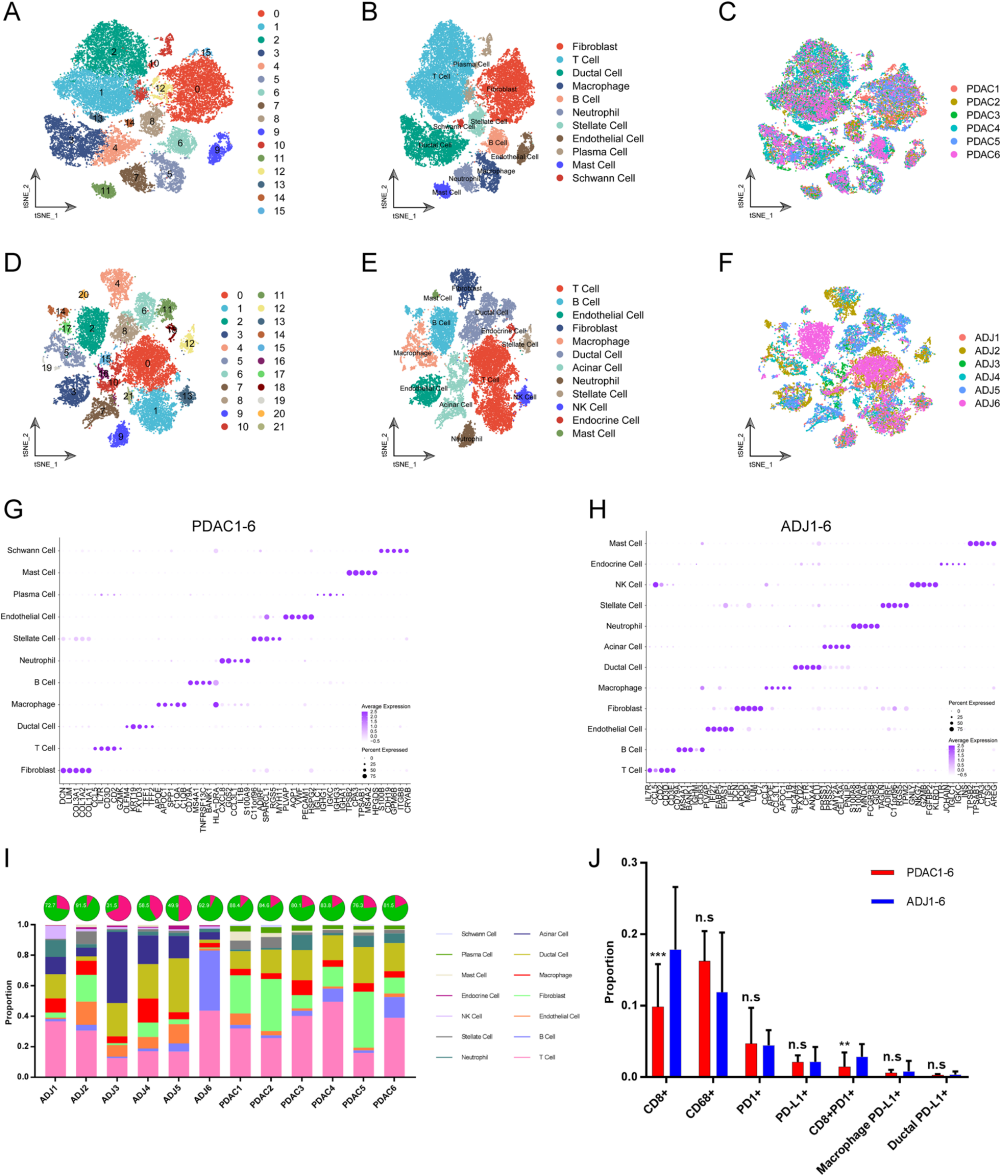
ScRNA-seq delineates the cellular landscape of PDAC and adjacent noncancerous tissues. **A**–**C** t-SNE plot showing original cell clusters (**A**), renamed cell types (**B**), specimens derived information in PDAC (C). Each dot represented one cell, and cell types were coded with different colors. **D**–**F** t-SNE plot showing the cellular landscape of adjacent noncancerous tissues. **G**–**H** Dot plots showing top 5 cell types-specific genes. Size of dots indicated the proportion of cells expressing the selected genes, and intensity of color (from white to purple) indicated the average expression level. **I** The box plots showing the proportion of cell types in PDAC and the counterpart tissues. For pie charts, the proportion of stromal cells (green) was showed. **J** The differences in the proportion of positive cells expressing immune marker between PDAC and adjacent noncancerous tissues

To further characterize the immune profiles and PD1/PD-L1 expression in PDAC at single-cell resolution, the six PDAC and six adjacent noncancerous tissues were obtained following pancreatectomy to conduct scRNA-seq. A large gene-cell matrix, consisting of 58,076 cells [32849 cells from the PDAC tissues and 25,227 cells from the adjacent noncancerous tissues (ADJ)] and 24,148 genes, was generated after stringent quality control using the CellRanger and Seurat tools. The t-distributed stochastic neighbor embedding (t-SNE) was used and cells were segregated into various clusters in two dimensions according to their transcriptional profiles. A total of 16 original clusters were identified in the PDAC group, whereas 22 original clusters were identified in the ADJ group (Fig. 6A, D). By cross-referencing the cluster-specific genes with known signatures of cell populations reported in previous studies [18, 19], these clusters were annotated as known cell types, including fibroblasts (LUM), T cells (CD3D), ductal cells (KRT19), macrophages (CD68), B cells (MS4A1), neutrophils (S100A8), stellate cells (RGS5), endothelial cells (CDH5), plasma cells (MZB1), mast cells (TPASB1), schwann cells (S100B), acinar cells (PRSS1), natural killer (NK) cells (FGFBP2), endocrine cells (CHGA) (Fig. 6B, E and Additional file 1: Fig. S1A–B). No specimen-specific cluster was found, indicating no significant specimen-derived batch effect (Fig. 6C, F). The top five cell-specific genes for each cell type were shown in Fig. 6G, H.

The cellular compositions of PDAC and ADJ tissues were calculated. We found that cellular compositions varied among these specimens, indicating inter-patient heterogeneity (Fig. 6I). A higher proportion of fibroblasts was found in PDAC specimens. In addition, there were more abundant stromal cells in the PDAC specimens compared to ADJ specimens (82.45 vs. 66.17, p < 0.0001), which was consistent with the result of the mIHC (Figs. 3B, 6I). Subsequently, we explored the previously studied-immune related features using scRNA-seq analysis. Consistent with previous findings, PDAC specimens had a lower proportion of CD8 + (CD8A) cells and CD8 + PD1 + (PDCD1) cells than ADJ specimens. However, no significant differences were detected in the proportion of CD68 + cells and PD1 + cells between the two groups (Fig. 6J). For PD-L1 (CD274) expression, we also did not find a statistical difference in total PD-L1 + cells, PD-L1 + macrophages, and PD-L1 + ductal cells (Fig. 6J).

To further investigate the distribution of PD1/PD-L1 in the PDAC and ADJ tissues at single cell transcriptomic level, the expression levels of selected genes in each cell were mapped to the t-SNE plots (Additional file 1: Fig. S2A–B). For both PDAC and ADJ specimens, CD68 and CD8A expression were restricted to the corresponding macrophages and T cells. The PDCD1 was mainly enriched in T cells. However, CD274 was expressed in a variety of cell types, including macrophages, ductal cells, and other immune cells. The proportion of CD274 + cells was relatively low, responding to the fact that PDAC is a cold tumor.

### The expression of PD1 and PD-L1 in T cell and macrophage subpopulations

**Fig. 7:**
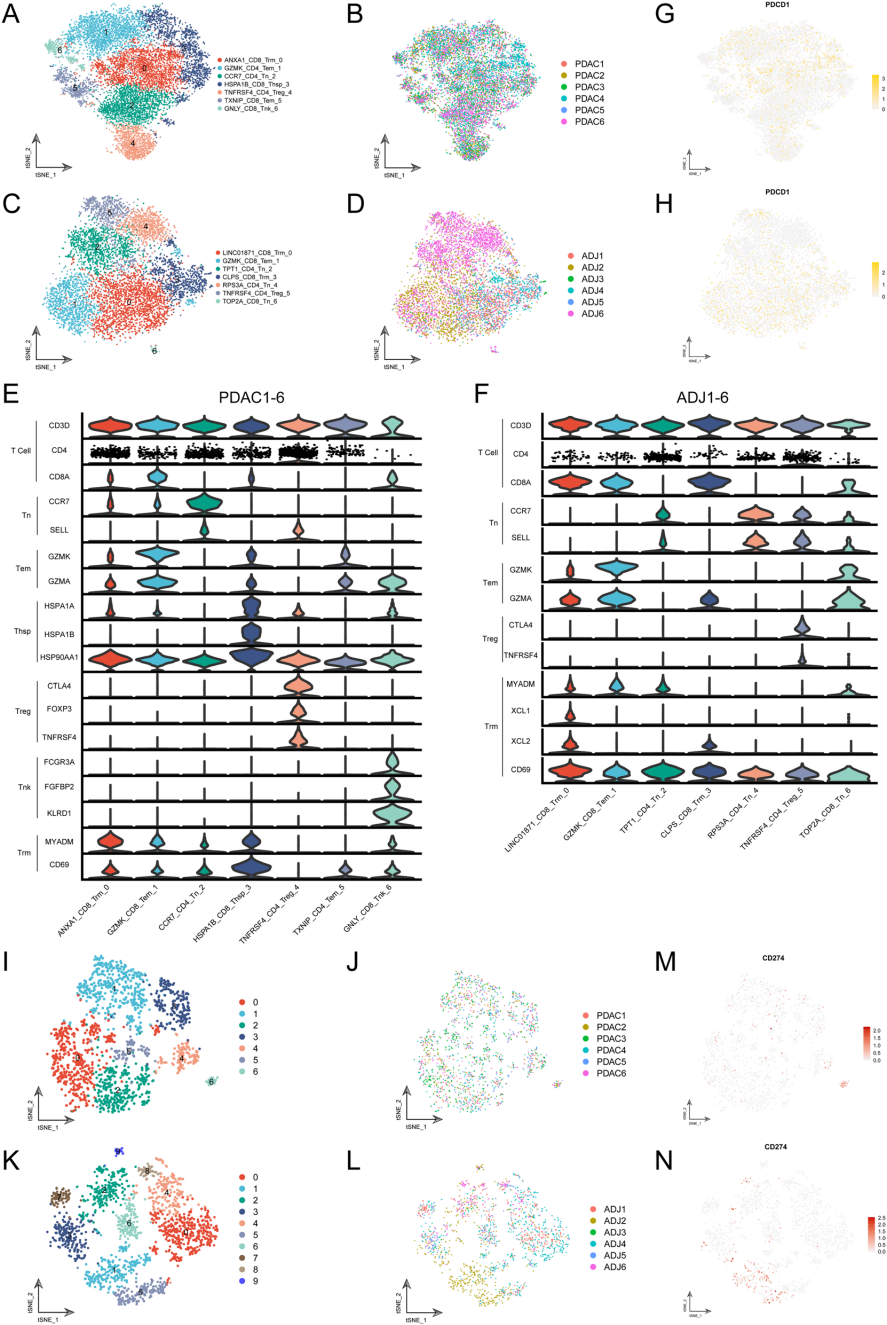
Immune profiles of PDAC and adjacent noncancerous tissues. **A**–**B** t-SNE plot showing renamed T cell subpopulations (**A**), specimens derived information in PDAC (**B**). Each dot represented one cell, and cell types were coded with different colors. **C**–**D** t-SNE plot showing the T cell subclustering analysis of adjacent noncancerous tissues. **E**–**F** Violin plots showing the normalized expression levels of known signature genes of distinct T cell subpopulations. **G**–**H** The distribution of PDCD1 + (PD1 +) T cells in PDAC and adjacent noncancerous tissues. The intensity of color (from white to yellow) indicated the average expression level of PDCD1. **I**–**J** t-SNE plot showing original cell cluster of macrophages (**I**), specimens derived information in PDAC (**J**). Each dot represented one cell, and cell types were coded with different colors. **K**–**L** t-SNE plot showing the macrophage subclustering analysis of adjacent noncancerous tissues. (**M**–**N**) The distribution of CD2741 + (PD-L1 +) macrophages in PDAC and adjacent noncancerous tissues. The intensity of color (from white to red) indicated the average expression level of CD274

To better understand the expression of PD1 and PD-L1 in the tumor microenvironment of PDAC, we isolated gene-cell matrixes of T cells and macrophages and conducted separate clustering analyses. The t-SNE plots showed that T cells were further grouped into seven and seven major unsupervised clusters in the PDAC and ADJ specimens, respectively (Fig. 7A, C), including naïve T (Tn), effector memory T (Tem), resident memory T (Trm), regulatory T (Treg), and NK-like T (Tnk). Remarkably, a new T cell type, which was characterized with expressing multiple heat shock protein (HSP) family members, such as HSPA1A, HSPA1B, and HSP90AA1 was identified, and therefore was named HSP T (Thsp). This T cell subpopulation was only observed in PDAC but not in the ADJ specimens (Fig. 7A, C). No specimen-specific T cell subpopulation was found (Fig. 7B, D). The identity of each T cell subpopulation was verified according to known cell markers (Fig. 7E–F). Next, we explored whether PD1 + cells were restricted to a particular T cell subpopulation. The expression level of PDCD1 was mapped to the t-SNE plot (Fig. 7G–H). The results showed that PDCD1 + cells spread across different T cell subpopulations, suggesting that anti-PD1 immunotherapy may affect all T cell subpopulations. We speculated that targeting the whole T cell population, rather than a specific T cell subpopulation (Treg), might contribute to the insensitivity of PDAC patients to anti-PD1 treatment.

A total of 7 and 10 major unsupervised macrophage subpopulations were identified in the PDAC and ADJ specimens, respectively (Fig. 7I, L). Each macrophage subpopulation was composed of cells from multiple specimens (Fig. 7J, M). The t-SNE plots showed that subcluster 6 in the PDAC group and subclusters 1 and 5 had enriched expression of CD274 (Fig. 7K, N). Therefore, the anti-PD-L1 drugs might mainly target specific macrophage subpopulations, while their roles in the tumor microenvironment and immunotherapy remain to be investigated.

### The identification of PD-L1 + malignant ductal cells

**Fig. 8:**
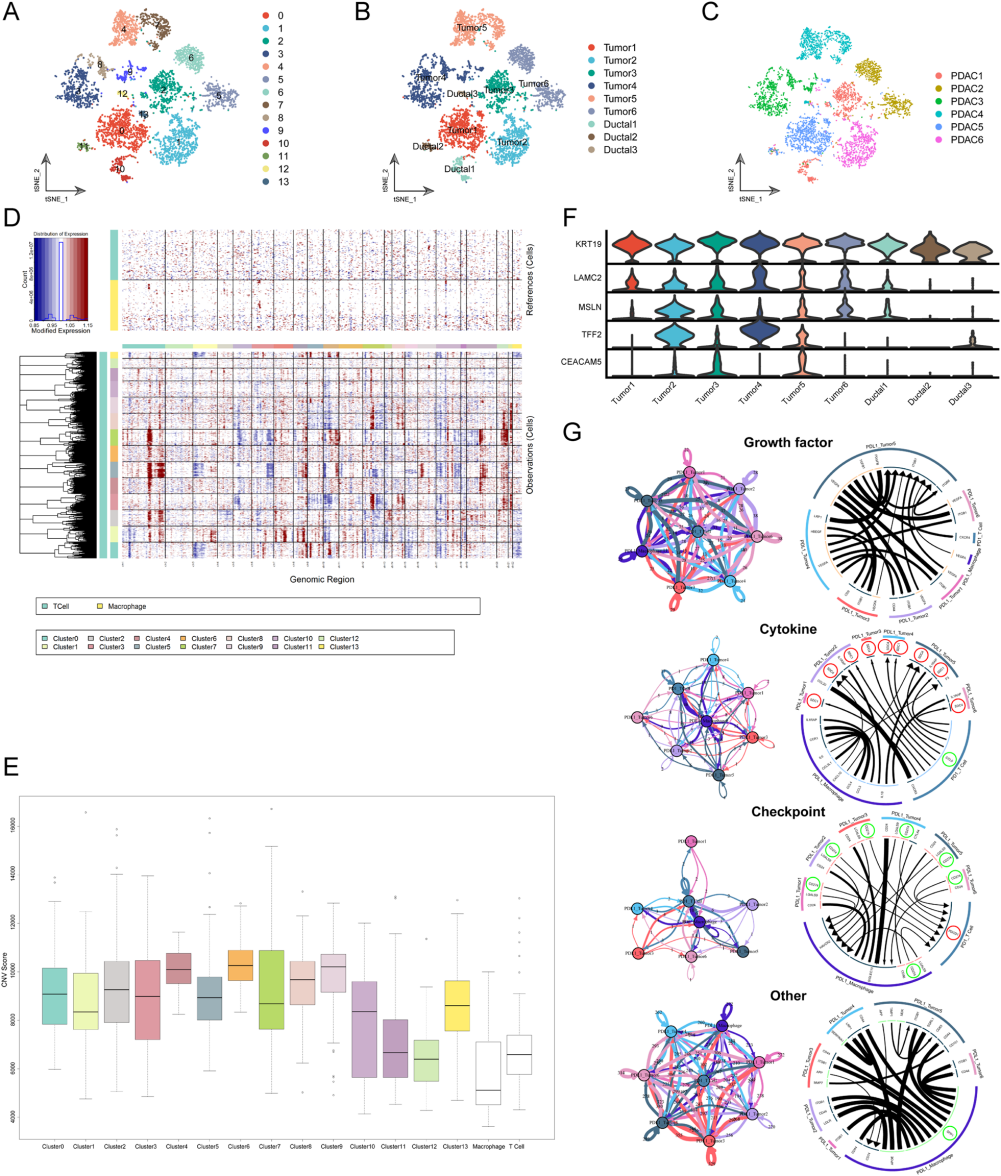
Receptor-ligand interaction between PD1 + T cells and PD-L1 + macrophages and tumor cells. (**A**–**C**) t-SNE plot showing original ductal cell clusters **A** renamed ductal cell subpopulations (**B**), specimens derived information in PDAC (**C**). Each dot represented one cell, and cell types were coded with different colors. **D** Heatmap showing large-scale CNV profile of each ducal cell cluster. Blue and red colors represented low and high CNV levels, separately. Both macrophages and T cells were used as reference cells. **E** Box plots were used to compare the CNV scores of distinct ductal cell clusters. White box represented reference cell clusters. **F** Violin plots showing the normalized expression levels of tumor marker genes among these ductal cell clusters. The expression of KRT19 was the common marker of ductal cells. **G** Chord diagram showing the receptor-ligand pairs among PD1 + T cells and PD-L1 + macrophages and tumor cells, which were classified into four categories, including growth factors, cytokines, immune checkpoints, and others. Connection graph in the left panel showing the intensity of interactions between two main cell clusters in colored circles. Total counts of ligand-receptor pairs are counted

Next, ductal cells were isolated to construct a new gene-cell matrix, and subclustering analysis was conducted. A total of 14 original ductal clusters were identified (Fig. 8A). To further isolate malignant ductal cells, large-scale chromosomal copy number variation (CNV) analysis was performed, and the CNV score of each cell was calculated. Both T cells and macrophages were taken as reference cells because they are considered to have no CNV. There were significantly higher CNV scores in the subcluster 0/1/2/3/4/5/6/7/8/9/13 than in the reference cells, therefore were defined as tumor clusters, while subcluster 10/11/12 had no obvious CNV, and were therefore defined as normal ductal clusters (Fig. 8B, D–E). Interestingly, cells from different PDAC specimens clustered separately, indicating inter-patient heterogeneity (Fig. 8C). Moreover, tumor clusters had higher expression levels of tumor makers, including LAMC2, MSLN, TFF2, and CEACAM5, compared with ductal clusters, which further verified their tumor identity (Fig. 8F). We compared PD-L1 + and PD-L1—tumor cells and found 417 differentially expressed genes (DEGs), which were enriched in the regulation of biosynthetic process, development process, and transcription regulator activity by GO analyses (Additional file 1: Fig.S3).

### Crosstalk between PD1 + T cells and PD-L1 + tumor cells

To gain insight into the cell-to-cell interactions between targeted cells of ICIs in PDAC, a new gene-cell matrix of PD1 + T cells and PD-L1 + tumor cells/macrophages was constructed, and the receptor-ligand interactions between them were inferred using the iTALK method. Strong connections were observed between these cells (Fig. 8G). The PD1 (PDCD1)-PD-L1 (CD274) receptor-ligand pairs were identified between PD1 + T cells and PD-L1 + tumor cells. Remarkably, we found that the chemokine CCL5 secreted by PD1 + T cells could combine with SDC1/4 on the surface of all PD-L1 + tumor clusters (Tumor1-6). In addition, there was an SPP1-ITGB1/CD44 receptor-ligand connection between PD-L1 + macrophages and tumor clusters (Fig. 8G). Therefore, the tumor microenvironment contains a complex cell-to-cell communication that might be a promising drug target for improving the efficacy of ICIs in PDAC.

## Discussion

PDAC is characterized by a complex tumor microenvironment, mainly consisting of tumor cells, endothelial cells, fibroblasts, and many different types of tumor infiltrating immune cells [20, 21]. There are sophisticated cell-to-cell communications between immune cells and tumor cells, which are involved in every step of tumor progression in PDAC [22, 23]. Tumor immune infiltration varied a lot among patients with PDAC, which was closely correlated to prognosis and response to immunotherapy [24]. Recently, the clinical significance of the immune checkpoint molecular PD1/PD-L1 has attracted widespread attention. Nevertheless, it remains challenging to reach a consensus regarding the cut-off value for determining whether a tumor is PD1/PD-L1 positive, and the prognostic value of PD-L1 was inconsistent in previous studies [25–27]. In this study, we captured new immune features using mIHC that were different from the traditional anatomical features for the TNM staging system. The expression levels of PD1/PD-L1 were determined by conducting quantitative pathological analysis. Compared to previous conventional IHC and RT-qPCR methods, this method was more objective and reproducible. Moreover, the tumor and stromal segmentations were performed so that we could further determine these features in different regions. The spatial features of PD1-PD-L1 interaction were also obtained. UniCox and MultiCox analyses were employed to develop a novel risk score-based prognostic model using immune features generated by mIHC and quantitative pathology. Finally, a nomogram plot was constructed by combining the risk score and vital clinicopathological characteristics, with important clinical significance for predicting the prognosis of patients with PDAC.

In prior studies, only a single marker was detected to evaluate its prognostic value. Here, we simultaneously measured multiple markers in the same section to characterize the immune landscape of PDAC. The co-expression and correlation of multiple markers were also determined. In addition, scRNA-seq analysis was conducted to further verify the results of the mIHC and quantitative pathological analyses. Overall, we demonstrated that PD-L1 expression at the RNA and protein levels was relatively scarce in PDAC, consisting with previous reports despite different methods were employed [28]. The lacking of PD-L1 might contribute to the ineffectiveness of ICIs. The mechanism of PD-L1 downregulation in the PDAC microenvironment remains to be clarified, which is vital for improving the effectiveness of anti-PD-L1 treatments.

Tumor cells escape immune surveillance and are insensitive to immunotherapy, mainly attributed to the activation of immune checkpoint molecules and the interactions between tumor cells and suppressive immune cells. Thus, ICIs, such as anti-PD1 drugs, might have a tumor-killing effect. However, a single agent has not yet yielded significant improvements in the prognosis of patients with PDAC [29]. It is now believed that exploring combination regimens is a promising direction. In this study, we found that PD1 + cells were distributed in different T cell subpopulations (Fig. 7G–H). Therefore, anti-PD1 treatments target not only Tregs, which play a negative regulatory role in the tumor microenvironment, but also other T cell subpopulations. Extensive inhibitory effects on T cell subpopulations might cause treatment failure. The MultiCox result showed that the proximity relationship between PD1 + CD8 + T cells and the PD-L1 + region was an independent prognostic factor in PDAC (Table 2), indicating the important clinical value of spatial information in the tumor microenvironment. However, the specific types of PD1 + T cell subpopulations close to the PD-L1 + region need to be further explored. Both mIHC and scRNA-seq analyses showed that PD-L1 (CD274) expression was low, while the proportion of stromal cells was relatively high, which explained why PD1/PD-L1 treatment failed in PDAC patients. Furthermore, we performed iTALK analysis and observed that there were other conserved receptor-ligand pairs (CCL5-SDC1/4) besides the PD1-PD-L1 interaction between PD1 + T cells and PD-L1 + tumor cells. It was reported that the combined blockade of PD-L1 and CCL5 synergistically suppressed tumor growth in xenograft and orthotopic PDAC mouse models [30]

The minority of patients with malignant tumors have a better response to ICIs, especially those with mismatch repair deficiency and high mutation burden. There is still no good way to screen for patients who will benefit from immune checkpoint treatments. Both mIHC and scRNA-seq could reveal immune profiles very well and identified novel features at unprecedented resolution. In this study, a risk score-prognostic model based on immune features generated by mIHC was developed, which showed a good performance in predicting overall survival of patients. In the future, new prediction system based on these methods might well compensate for deficiencies of mismatch repair deficiency and mutation burden in predicting immune checkpoint treatment response.

This study has several limitations. First, the limited sample size was used to conduct mIHC on TMA, and there was no external validation for the risk score-based prognostic model using immune features. Second, TMA had an inevitable deficiency in that a small TMA core area tended to lead to sampling bias. However, TMA cores from different patients were stained with multiple markers simultaneously, which reduced the batch effect caused by multiple staining of traditional IHC using serial sections. Third, it is necessary to stain for more markers to further characterize the immune profiles of PDAC. Here, we focus only on the important immune checkpoint molecules PD1/PD-L1. Finally, there was a preference for the preparation of single cell suspensions during tissue dissociation, which might cause errors in cell composition analysis to some extent. In addition, scRNA-seq was less friendly to some molecules with low expression, thus it might underestimate the expression of PD-L1 (CD274) in PDAC.

## Conclusions

Overall, our work reveals the immune landscape of PDAC by combining mIHC and scRNA-seq analyses. A novel risk score-based prognostic model using immune features generated by quantitative pathology was developed. We found other conserved receptor-ligand pairs between PD1 + T cells and PD-L1 + tumor cells, which might provide new clues for detecting targets in combination with ICIs. Further studies will be required to reveal the immune landscape, tumor microenvironment heterogeneity, and underlying molecular mechanism, which will facilitate developing new immunotherapy combination strategies for PDAC.

## Supplementary Information

**Additional file 1: Figure S1.** Identification of cell types. **A**–**B** Violin plots showing the normalized expression levels of known signature genes of distinct cell populations in PDAC (**A**) and adjacent noncancerous tissues (**B**). **Figure S2.** Expression and distribution of immune markers. **A**–**B** The distribution of CD68+, PDCD1+ (PD1+), CD2741+ (PD-L1+), CD8A+ (CD8+) cells in PDAC (**A**) and adjacent noncancerous tissues (**B**). The intensity of colors (from white to specific colors) indicated the average expression level of immune markers. **Figure S3.** Gene annotation analysis of differentially expressed genes (DEGs) between PD-L1+ and PD-L1- tumor cells. Biological process (**A**), and Molecular function (**B**). Top GO terms were shown. Bar plots are colored according to their − log10P-values. Heatmaps showing the expression levels of top 80 DEGs.

**Additional file 2: Table S1.** Clinical characteristics of PDAC patients.

**Additional file 3: Table S2.** Features of TMA cores generated by Qupath.

## Data Availability

The scRNA-seq dataset for the PDAC and adjacent noncancerous specimens has been deposited in Gene Expression Omnibus (GEO) repository with Accession Number: GSE212966 (PRJNA879876). All related codes and data analysis scripts will be provided upon request to first author Kai Chen (Drchenkai@pku.edu.cn).
