# Supplementary material for: Immune profiling and prognostic model of pancreatic cancer using quantitative pathology and single-cell RNA sequencing
Source: J Transl Med. 2023 Mar 21;21:210. doi: 10.1186/s12967-023-04051-4 (PMC10031915; doi:10.1186/s12967-023-04051-4)
Supplement: Supplementary file 1 — Additional file 1: Figure S1. Identification of cell types. A–B Violin plots showing the normalized expression levels of known signature genes of distinct cell populations in PDAC (A) and adjacent noncancerous tissues (B). Figure S2. Expression and distribution of immune markers. A–B The distribution of CD68+, PDCD1+ (PD1+), CD2741+ (PD-L1+), CD8A+ (CD8+) cells in PDAC (A) and adjacent noncancerous tissues (B). The intensity of colors (from white to specific colors) indicated the average expression level of immune markers. Figure S3. Gene annotation analysis of differentially expressed genes (DEGs) between PD-L1+ and PD-L1- tumor cells. Biological process (A), and Molecular function (B). Top GO terms were shown. Bar plots are colored according to their − log10P-values. Heatmaps showing the expression levels of top 80 DEGs. [file 12967_2023_4051_MOESM1_ESM.pdf]

Supplemental Text and Figures

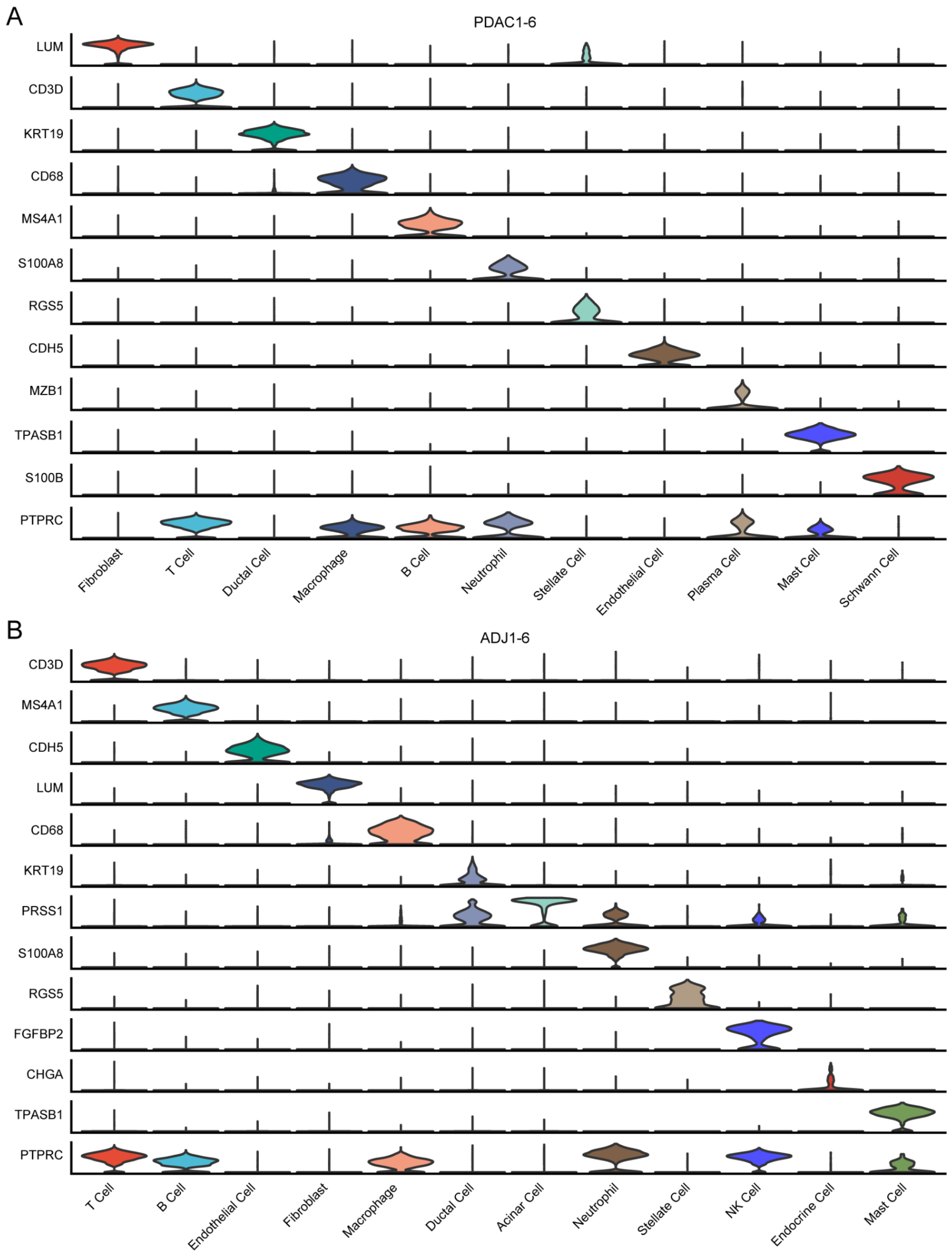

Fig.S1 Identification of cell types. (A-B) Violin plots showing the normalized expression levels of known signature genes of distinct cell populations in PDAC (A) and adjacent noncancerous tissues (B).

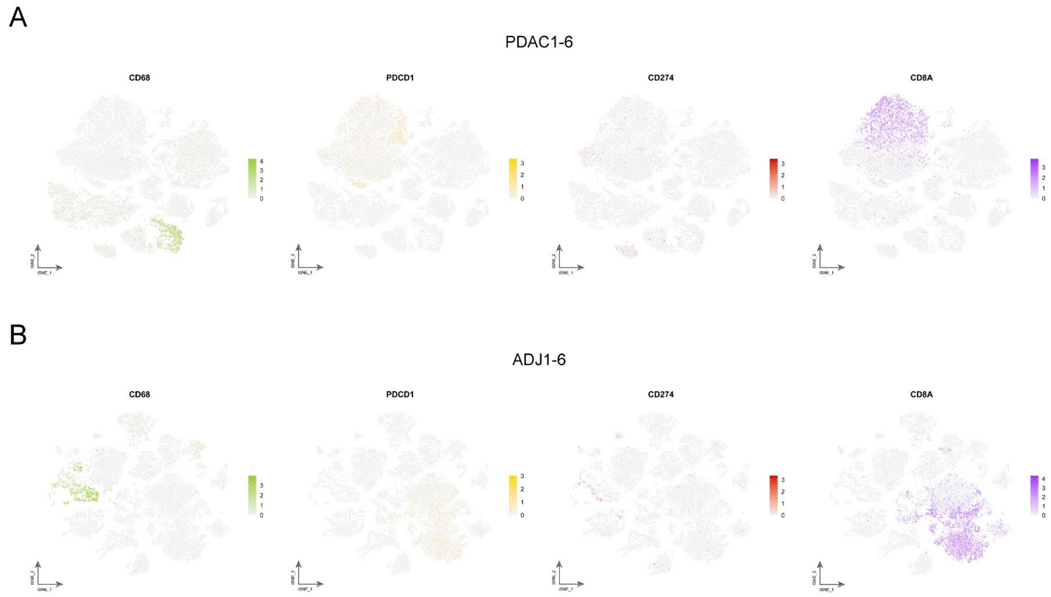

Fig.S2 Expression and distribution of immune markers. (A-B) The distribution of CD68+, PDCD1+ (PD1+), CD2741+ (PD-L1+), CD8A+ (CD8+) cells in PDAC (A) and adjacent noncancerous tissues (B). The intensity of colors (from white to specific colors) indicated the average expression level of immune markers.

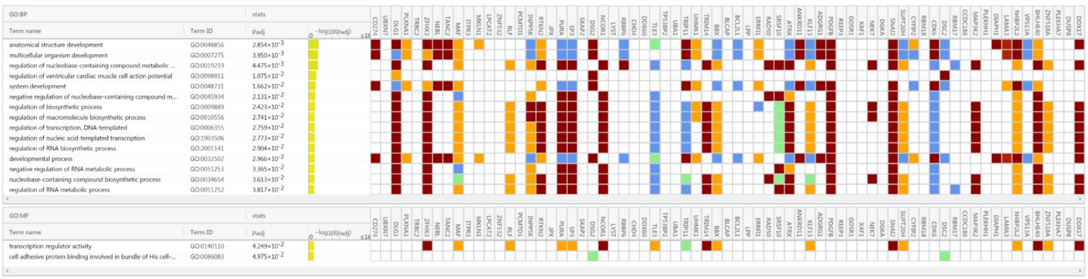

Fig.S3 Gene annotation analysis of differentially expressed genes (DEGs) between PD-L1+ and PD-L1- tumor cells. Biological process (A), and Molecular function (B). Top GO terms were shown. Bar plots are colored according to their -log10P-values. Heatmaps showing the expression levels of top 80 DEGs.
